# Extracting unresolved coupling constants from complex multiplets by a real‐time J‐upscaled SERF experiment

**DOI:** 10.1002/mrc.4699

**Published:** 2018-01-07

**Authors:** Kathrin Buchberger, Martin Walenta, Klaus Zangger

**Affiliations:** ^1^ Institute of Chemistry/Organic and Bioorganic Chemistry University of Graz Heinrichstrasse 28 A‐8010 Graz Austria

**Keywords:** homonuclear broadband decoupling, interrupted acquisition, NMR spectroscopy, real‐time J‐upscaling, scalar coupling, selective refocusing, spatially selective excitation, structure analysis

## Abstract

The measurement of small homonuclear coupling constants is often prevented by either their small size and/or overlap with other signal splittings. Here, we present a real‐time method to extract such couplings without interference from other splittings, with a resolution that is beyond conventional NMR spectra. In this real‐time J‐upscaled SERF experiment, homonuclear coupling is removed by slice‐selective pure shift NMR, whereas scalar coupling to only one selected signal is reintroduced by selective refocusing. The remaining couplings are enhanced by real‐time J‐upscaling during interruptions of the FID data acquisition. The resulting spectrum is not only simplified by the restriction of the scalar coupling but also its resolution enhanced. This improved resolution results from a reduction of signal broadening due to magnetic field inhomogeneities from 2 different sources: slice‐selective excitation and the spin‐echo type J‐upscaling element.

## Graphical abstract

The real‐time J‐upscaled selective refocusing experiment allows the extraction of small coupling constants, even when they are overlapped by other splittings. All homonuclear couplings are removed by slice‐selective pure shift NMR, whereas scalar coupling to only one selected signal is reintroduced by selective refocusing. The remaining couplings are enhanced by real‐time J‐upscaling during interruptions of the FID data acquisition. The resulting spectrum is not only simplified by the restriction of the scalar coupling but also its resolution enhanced.

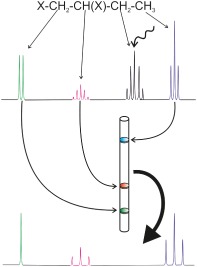

## 1 INTRODUCTION

The main parameters extracted from NMR spectra and used for the structural analysis of organic and biomolecules are the chemical shift and scalar coupling constants. Due to its highest sensitivity, ^1^H NMR is most often used when the compounds to be studied are at natural isotopic abundance. However, the resolution of protons is rather low compared to other nuclei, which results from a narrow chemical shift range and extensive homonuclear scalar coupling. Although homonuclear proton‐proton coupling constants contain important structural information about molecular connectivities and dihedral angles, they also significantly contribute to spectral crowding. To lower the chances of signal overlap and thereby increase the resolution of NMR spectra, the removal of homonuclear scalar couplings by pure shift NMR has been suggested.^1^, ^2^, ^3^, ^4^, ^5^, ^6^, ^7^, ^8^, ^9^, ^10^, ^11^, ^12^ In these spectra, all homonuclear couplings are removed, which yields singlet only spectra, reminiscent of ^1^H‐decoupled ^13^C NMR spectra. Although these experiments provide a good signal dispersion, resulting in high resolution NMR spectra as far as chemical shifts is concerned, scalar coupling information, which is often key in analyzing chemical structures, is of course, completely lost. A number of alternative experiments, which make the extraction of scalar coupling information easier, have been proposed, such as two‐dimensional E‐COSY type spectra,^13^ soft‐COSY^14^, or the MUSIC experiment.^15^ For complicated multiplets or larger molecules, these spectra still often result in crowded multiplet patterns. One technique to significantly simplify scalar coupling patterns and therefore enable the extraction of individual couplings is the selective refocusing (SERF) experiment proposed by Fäcke and Berger.^16^ In the original version, two coupling partners are selectively excited and only their mutual scalar coupling evolves during t_1_, resulting in a two‐dimensional *J*‐resolved experiment showing the splitting between the two selected spins only. This method allows the unperturbed determination of scalar coupling constants but it would be impractical for a larger number of coupling constants, as n(n‐1) two‐dimensional experiments are required to measure all coupling constants in a system of n coupled signals. Several extensions and improvements of the basic SERF experiment have been described and applied to several systems.^17^, ^18^, ^19^, ^20^ Recently, the spatially separated, parallel acquisition of all SERF spectra connected to one selected peak has been described by Giraud et al.^21^ For this experiment, a selective pulse is applied during a weak pulsed field gradient, resulting in the excitation of the whole spectrum, but different signals are selected in different slices of the sample tube. Subsequently, a spatially selective 2D SERF spectrum is recorded, where each signal stems from a different slice of the NMR sample.^19^, ^22^, ^23^, ^24^, ^25^, ^26^ Recently, a combination of PSYCHE decoupling in F_2_ with a 2D SERF was described by Sinnaeve et al.^27^ Coupling constants are extracted from the resulting 2D *J*‐resolved spectrum. The gain in resolution in the spatially selective 2D SERF variant comes at the price of a significantly reduced sensitivity because each signal originates in a narrow slice of the sample tube.^28^, ^29^, ^30^ Combined with the need to record 2D spectra for all these SERF experiments, long measurement times are usually required. Recently, both Suryaprakash et al.^24^, ^31^, ^32^, ^33^ and Gubensäk et al.^34^ independently described a one‐dimensional, real‐time version of the SERF experiment, which allows the recording of one‐dimensional spectra, showing all scalar couplings to one selected signal only. The real‐time SERF experiment is essentially a slice‐selective (Zangger–Sterk) pure shift spectrum,^7^ where scalar coupling to one selected signal only is allowed to evolve during the chunked FID. Signals not directly coupled to the selected spin are reduced to singlets. Limiting the scalar coupling evolution during acquisition is achieved by spatially selective *J*‐coupling refocusing during interrupted acquisition.^6^ Although the real‐time SERF experiment allows the unperturbed observation of individual coupling constants between two signals, the resolution limit is still the signal linewidth. Recently, we have shown that very small scalar couplings, which are hidden within the NMR linewidth, can sometimes be made visible by real‐time *J‐*upscaling,^35^, ^36^ where the FID is again acquired in a real‐time chunked mode. During the acquisition interruptions, scalar coupling is allowed to evolve whereas chemical shift evolution is refocused. The result is a spectrum where the chemical shifts are unperturbed, and all couplings are enlarged by the same factor, which depends on the relative durations of the FID data chunks and the interruptions. However, the coupled signals are not only expanded. The scalar coupling resolution is effectively enhanced because any signal broadening by magnetic field inhomogeneities is removed during the interruptions by a central 180° pulse. Even on well shimmed state‐of‐the‐art spectrometers, this leads to a significant scalar coupling resolution enhancement when small *J* values (below 2 Hz) need to be extracted.^35^ It should however be noted that the term “*J*‐scaling”, which has been used historically for such experiments^37^, ^38^ is actually misleading. It is, as a matter of fact, a downscaling of chemical shift evolution, which in combination with longer evolution times, yields spectra with enhanced scalar coupling, relative to chemical shift evolution.

## 2 THEORY

**Figure 1:**
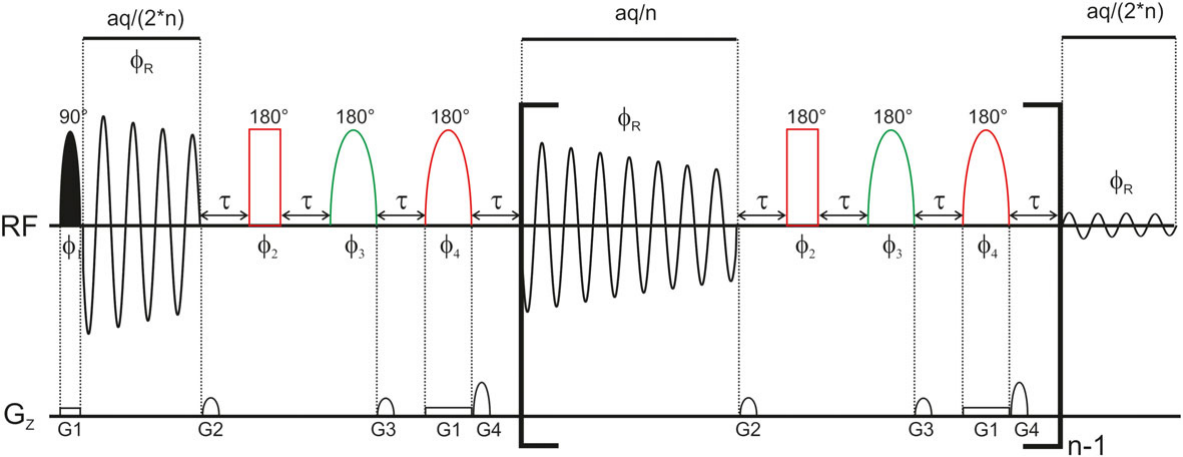
Pulse‐sequence of the *J*‐upscaled real‐time SERF experiment. Selective pulses are represented by half‐ellipsoids. During the acquisition interruption, a combination of slice‐selective pure shift NMR (red pulses) is combined with selective refocusing (green pulse) and real‐time J‐upscaling (during 2τ). The total acquisition time is aq and the pulse phases are ϕ_1_ = ϕ_R_ = x,‐x,‐x,x,y,‐y,‐y,y; ϕ_2_ = x,‐x; ϕ_3_ = x,x,‐x,‐x; ϕ_4_ = ‐x,x. The relative gradient strengths are G_2_:G_3_:G_4_ = ‐1:1:2. The strength of G_1_, which is the gradient for slice‐selective excitation, depends on the spectral width. It is typically ~1–2 G/cm

Here, we present a combination of slice‐selective pure shift NMR with SERF and real‐time *J*‐upscaling. The result is a one‐dimensional proton NMR spectrum showing enlarged scalar coupling to one selected signal only. Thereby, it is possible to measure coupling constants, which are too small to be observable in conventional NMR spectra and overlapped by other couplings. The pulse sequence for this real‐time *J*‐upscaled SERF experiment is shown in Figure 1. After a slice‐selective 90° pulse (a selective pulse applied during a weak pulsed field gradient), the acquisition of the first FID data block is started. Between successive FID chunks, scalar coupling evolution is refocused by a spatially selective 180° pulse together with a nonselective 180° pulse, similar to slice‐selective homonuclear broadband decoupling (also called Zangger–Sterk or ZS method). However, scalar coupling to one selected proton is allowed to evolve by an additional 180° pulse applied to the frequency of the desired coupling partner. For real‐time *J*‐upscaling, we introduce additional delays during the interruption. These delays need to be placed in a way that for the selectively refocussed coupling partners, no chemical shift evolution takes place, but additional scalar coupling evolution is present during the interruption. This is achieved by placing two delays symmetrically around the two pulses used for the pure‐shift part. These pulses both act on the observed, upscaled signals, but only the nonselective 180° pulse also on the coupling partner. Therefore, scalar coupling evolution is active during 2τ, whereas chemical shift evolution is refocused during the interruption. Due to better inversion performance over a large spectral range, we used a 180° CHIRP adiabatic pulse.^39^ The result is a 1D ^1^H spectrum, which shows only the active coupling to the selectively refocused signal upscaled by a tuneable factor λ, which depends on the length of the FID chunking duration and τ according to λ=(2τ+cd)/ cd, where cd is the chunk duration. As with other interrupted acquisition NMR experiments,^6^, ^40^, ^41^, ^42^ the interruptions are not visible in the detected FID if relaxation during the interruption is not too significant. The resulting FID can be processed like any conventional FID and does not need any sophisticated signal processing.

## 3 RESULTS AND DISCUSSION

**Figure 2:**
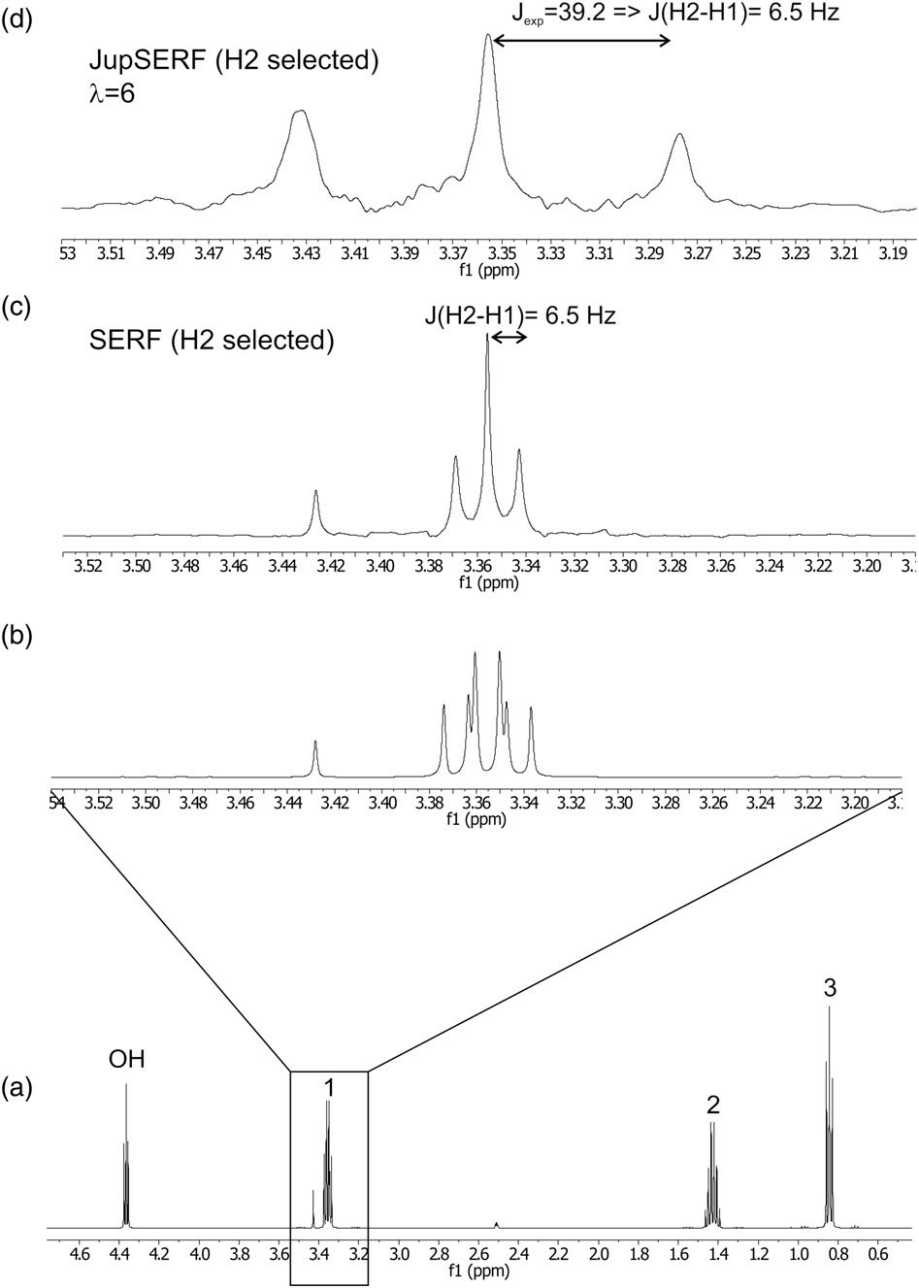
A conventional one‐dimensional ^1^H NMR spectrum of n‐propanol is shown in (a) and expanded in (b), together with a real‐time SERF spectrum of the H1 proton after selective refocusing of proton 2 in (c). A real‐time *J*‐upscaled SERF of the same proton with a scaling factor of six is shown in (d). From the apparent splitting of 39.2 and the scaling factor λ = 6, a coupling constants of 6.5 Hz is calculated. The signal at 3.43 ppm is from residual HDO (partly deuterated water). Eighty milligrams of propanol were dissolved in 500 μl DMSO‐d_6_. Thirty‐two scans were acquired for each of the spectra. The FID chunking time was 27 ms. A 40 ms E‐BURP pulse was used for excitation and 7 ms 180° Gaussian pulses were used for both homonuclear decoupling and selective refocusing. A 1 ms CHIRP 180° pulse was used for nonselective inversion during the interruption

As a first proof of concept of the presented method, a real‐time *J*‐upscaled SERF spectrum of n‐propanol can be seen in Figure 2. The resulting spectrum yields a significantly simplified scalar coupling pattern, which shows only the coupling to the selected resonance, upscaled by the factor indicated. Therefore, coupling constants can be extracted much more easily and accurately. The protons of the CH_2_‐group at Position 1 yield a double triplet (a doublet from the coupling to the OH group and a triplet from the other neighboring CH_2_ group) in the conventional NMR spectrum (Figure 2a,b) with a “coupling constant” of ~6.6 Hz for the triplet part. It is reduced to a pure triplet in the SERF spectrum (Figure 2c) using SERF of the H2 signal, with a coupling constant of 6.5 Hz. Overlap with the additional coupling to the OH group obviously led to a slight overestimation of this value. In the real‐time *J*‐upscaled SERF spectrum (Figure 2d), the coupling constant is enhanced sixfold, resulting in a splitting of 39.2 Hz, which corresponds to an actual *J* value of 6.5 Hz. The signal width at half height is 1.05 Hz in the regular spectrum, 1.10 Hz in the SERF spectrum, and 4.0 Hz for the sixfold upscaled spectrum, corresponding to 0.67 Hz when compared on the same scale. This corresponds to an actual resolution enhancement of the signal splittings by a factor ~1.6. The achievable resolution enhancement of real‐time *J*‐upscaling depends mainly on the residual magnetic field inhomogeneity and the potential presence of long‐range couplings. For the applications shown here, extensive shimming has been employed. In more routine service environments, the achievable scalar coupling resolution enhancement by *J*‐upscaling is probably even larger. A more detailed discussion of the resolution enhancement of *J*‐upscaling can be found in Glanzer and Zangger.^35^ The sensitivity of real‐time SERF spectra is of course markedly reduced compared to regular 1D spectra as a result of spatially selective excitation. The loss in sensitivity depends mainly on the required spectral width and the selectivity of the decoupling pulse. For a 10 ppm range and a 10 ms Gaussian pulse, the total signal intensity of the selectively refocused spectrum is about 3% of a regular 1D spectrum. However, some of this loss is regained by the partial removal of couplings and the narrower signals, which result from the increased magnetic field homogeneity in a single slice as compared to the whole detection volume. Real‐time *J*‐upscaling also slightly reduces the sensitivity due to the expansion of the coupled signals, but to an extent much smaller than slice‐selective excitation.^35^

**Figure 3:**
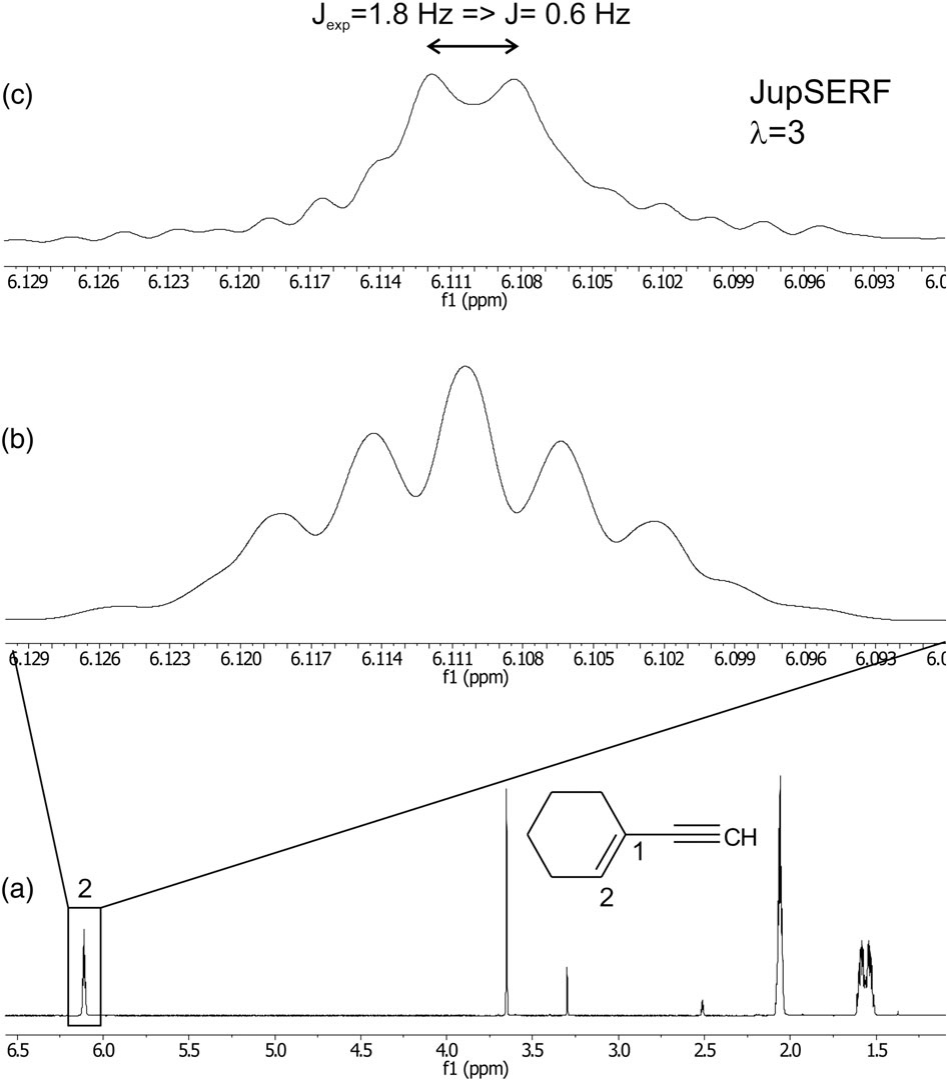
A conventional one‐dimensional ^1^H NMR spectrum of 1‐ethynyl‐cyclohexene is shown in (a) and the H2 proton signal expanded in (b). This signal shows a complicated multiplet due to couplings to other protons in the cyclohexene moiety and rather broad lines. A threefold upscaled real‐time SERF spectrum is shown in (c) after selective refocusing of the side‐chain methine proton. From the apparent splitting of 1.8 Hz and the scaling factor λ = 3, a *J* value of 0.6 Hz is calculated for this five‐bond coupling. In the conventional spectrum, this splitting is not visible and only leads to relatively broad lines of the multiplet. Eighty milligrams of 1‐ethynyl‐cyclohexene were dissolved in 500 μl DMSO‐d_6_. Thirty‐two scans were acquired for each of the spectra. The FID chunking time was 27 ms. A 40 ms E‐BURP pulse was used for excitation and 7 ms 180° Gaussian pulses were used both for homonuclear decoupling and selective refocusing. A 1 ms CHIRP 180° pulse was used for nonselective inversion during the interruption

Although in the case of n‐propanol, more accurate coupling constants can be obtained with *J*‐upscaled SERF spectra; in other cases, a small coupling might be hidden in the signal linewidth and additionally overlapped by other couplings, which would render the extraction of *J* values completely impossible by conventional one‐dimensional NMR techniques. The 1D proton spectrum of 1‐ethynyl‐cyclohexene is an example (Figure 3). The methine proton is five bonds away from the CH proton at Position 2. In the conventional ^1^H NMR spectrum, no coupling between these two protons is visible. Proton H2 only shows couplings to the aliphatic protons of the cyclohexene moiety. In a real‐time *J*‐upscaled SERF experiment, with the SERF pulse set on the methine signal at 3.6 ppm and using threefold upscaling, a doublet becomes visible at proton H2 at 6.1 ppm. The measured coupling of 1.8 Hz corresponds to a regular coupling constant of 0.6 Hz. The signal splitting can be manipulated by variation of the chunking duration or interruption delay τ. By variation of the upscaling factor λ between two and four, we obtained back‐calculated *J* values for the five‐bond coupling between the methine and H2 proton of 0.6 ± 0.2 Hz, which is unobservable in the regular ^1^H NMR spectrum. The real‐time *J*‐upscaled SERF experiment thus enables the measurement of very small couplings, even when they are overlapped with other splittings. It enables highest resolution, unperturbed measurements of small coupling constants by simple visual inspection of a one‐dimensional spectrum. The improved resolution of the *J*‐values results from not only the removal of unwanted couplings by a pure‐shift type experiment but also the reduction of magnetic field inhomogeneity broadening. Two key elements of this experiment make it less susceptible to magnetic field inhomogeneity: (a) the slice‐selective excitation used for homonuclear broadband decoupling selects only a narrow slice where the magnetic field is much more uniform compared to the whole detection volume and (b) the chemical shift refocusing during real‐time *J*‐upscaling also refocuses magnetic field induced frequency differences.^35^ The result offers, to the best of our knowledge, the highest resolution one‐dimensional NMR experiment for scalar coupling constant determination, which is presently available. It should however be mentioned that transverse relaxation losses are amplified by the upscaling block. Therefore, its use on slowly tumbling large molecules, such as structured proteins, is limited.^43^ In this case, a pseudo 2D version, similar to the recently reported J‐PSHIFT experiment,^44^ would be more suitable. Another limitation of 1D SERF experiments in general is the fact that the signal selected for refocusing needs to be isolated in the spectrum. In cases where it is overlapped, a previous TOCSY step could be included.^32^

In conclusion, real‐time *J*‐upscaled SERF spectra allow the straightforward measurement of small coupling constants that are hidden in the linewidth of conventional NMR spectra and overlapped by other splittings. Only couplings to one selected signal are visible in the spectrum, whereas all others are eliminated by homonuclear broadband decoupling. A significant enhancement of resolution of *J*‐values is achieved by reducing magnetic field inhomogeneities due to a spin‐echo type sequence during *J*‐upscaling and slice‐selective excitation during the pure shift element.

## 4 EXPERIMENTAL

All chemicals were purchased from Sigma‐Aldrich (St. Louis, MO, USA) at >98% purity. All experiments were carried out on a Bruker AVANCE III 500 MHz spectrometer using a 5 mm TXI probe with z‐axis gradients at 300 K. One‐dimension real‐time *J*‐upscaled SERF spectra were obtained using a 40 ms EBURP‐2 pulse for slice‐selective excitation during a weak pulsed field gradient of 1.0 G/cm. During the interrupted acquisition, we used 7 ms 180° Gaussian pulses for refocusing and also for selective recoupling. Fifty individual FID data chunks were assembled automatically during acquisition, resulting in a total acquisition time of ~1.5 s. The upscaling delay τ was varied to obtain the desired scaling factor.
